# Study on the Fauna of Aquatic Insects in Northwestern Iran

**DOI:** 10.18502/jad.v14i1.2698

**Published:** 2020-03-31

**Authors:** Madineh Abbasi, Sara Doosti, Hassan Vatandoost, Nasibeh Hosseini-Vasoukolaei

**Affiliations:** 1 Department of Medical Entomology and Vector Control, School of Public Health, Tehran University of Medical Sciences, Tehran, Iran; 2 Department of Communicable diseases, Vice Health, Tabriz University of Medical Sciences, Tabriz, Iran; 3 Department of Medical Entomology and Vector Control, Medical School, Zanjan University of Medical Sciences, Zanjan, Iran; 4 Department of Environmental Chemical Pollutants and Pesticides, Institute for Environmental Research, Tehran University of Medical Sciences, Tehran, Iran; 5 Department of Medical Entomology and Vector Control, Health Science Research Center, Faculty of Health, Mazandaran University of Medical Sciences, Sari, Iran

**Keywords:** Aquatic insects, Fauna, Azerbaijan, Iran

## Abstract

**Background:**

Aquatic insects include groups of arthropods which at least one step of their life happens in water. Some of these insects play an important role in the transmission of various diseases to human and animals. Because there is a little information about the fauna of aquatic insects in Iran, this study was aimed to collect and identify aquatic insects in northwestern Iran.

**Methods:**

A descriptive cross sectional study was performed in July 2017 in Rivers of three villages of Osku County of East Azerbaijan Province, northwestern Iran. The specimens were collected using different methods including D frame net-collector, standard mosquito dipper, Sweep Netting and plastic pipette. The collected specimens were identified based on the systematic keys of aquatic insects.

**Results:**

Totally 285 samples were collected. Four orders including Diptera, Hemiptera, Ephemeroptera and Coleoptera were identified. Collected samples belonged to seven families of Tipulidae, Chironomidae, Corixidae, Culicidae, Hydrophilidae, Baetidae and Dytiscidae. The most predominant family was Culicidae belonging to order Diptera. Culiseta *longiareolata* was the most frequent species collected in the study area.

**Conclusion:**

Aquatic insects usually play an important role in the food chain of animals and some species of them act as predators and play an important role in the biological control of vectors. Therefore, more studies are required to carry out in the field of aquatic insects.

## Introduction

Aquatic insects include groups of arthropods which at least one steps of their life happens in water. About more than 30000 species of aquatic insects were identified which can live in the freshwater and some species are living in the brackish water (1). According to the fossil records, aquatic insects appeared in the Triassic (2) more than 150 million years after the appearance of insects. The most important places for aquatic insects are various and include shallow holes with stagnant water, pools and floodgates, large and small rivers, streams, beaches, lakes, mineral water and drinking water pools (3). Several of them live near the water and their life cycle can be completed in or depended to water. Therefore, these insects called semi aquatic insects (4). Many of these insects spend their primary stages in the water while the adults are completely terrestrial, for example the order of Ephemeroptera (Mayflies), Odonata (Dragonflies and Damselflies), Plecopter (Stoneflies), Diptera (Flies), Trichopter (Caddisflies). The adults lay their eggs singly or patches in or around the water (5). Some of these insects play an important role in the transmission of various diseases to human and animals such as dengue virus, Zika virus, West Nile virus, encephalitis, malaria, filariasis and other arboviral diseases (6, 7). Some of diseases are transmitted via biological or mechanical pathway to human by Tabanidae and Simuliidae (8). Some of them like dragonflies and damselflies can be the host of Termatodes (9). A few of them cause the mental annoyance and dermal damage on the human and animal hosts by their painful bite (10). Some aquatic insects play the role of contamination indicators of water (for example Ephemeroptera, Plecoptera and Trichoptera) and are mentioned as “biological indicators” of water quality (11, 12). Many of them are the main food supply for fishes and amphibians (13–16). Because there is little information about the fauna of aquatic insects in Iran, this study was aimed to collect and identify the aquatic insects in the northwestern Iran in order to open a new window to the vast aquatic insects of the world.

## Materials and Methods

### Study area

**Fig. 1.**
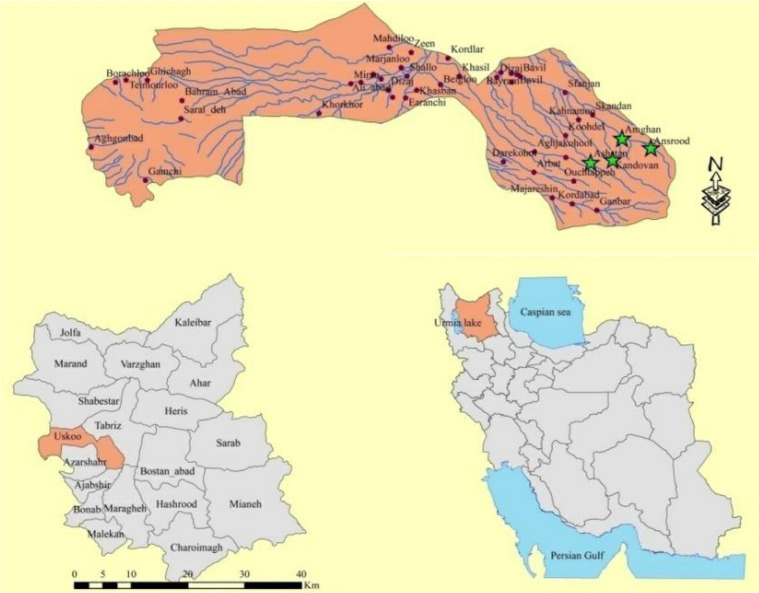
Geographical status of sampling sites in the study area in Osku County of East Azerbaijan Province, Iran

A descriptive cross sectional study was performed in July 2017 in the rivers of three villages of Osku County (Amghan, Ansrood and Kandowan). This county is one of 16 counties of East Azerbaijan Province of Iran located in the vicinity of Tabriz. The county with the geographical coordinates of 37° 51′ 29.54″ N, 45° 56′ 24.18″ E is located on the northwest of Sahand mountain range at an altitude of 1579 meters. Based on the results of the general census of population and housing in 2011, the population of the county of Osku is about 98,988 people (2.7% of the province’s population) and the population of the center of this county is estimated as 16983 people. Like other counties in Azerbaijan area, it has a short, mild summer and cold and long winters. Its precipitation is mostly in the cold seasons and its summers are dry and sometimes rainy (17) (Fig. 1)

### Sampling methods

**Fig. 2.**
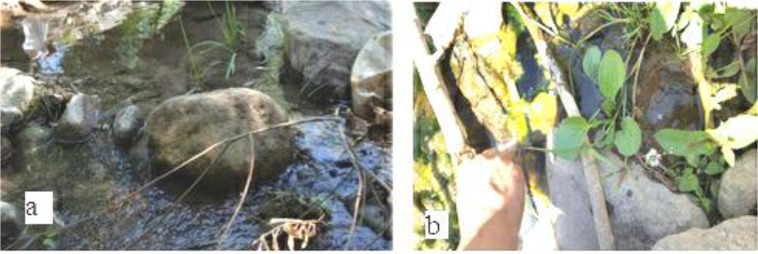
(a, b) Two sampling sites, Aghbolagh River, East Azerbaijan, Iran

The specimens were collected using different methods including: D frame net-collector, standard mosquito dipper (350ml), Sweep Netting and plastic pipette. Sampling carried out in different part of breading places in several occasions (Fig. 2). The samples were collected, transferred to individual jars containing some water obtained from their habitat. Subsequently they were put in glass vials contained 90% ethylic alcohol. The date and location of sampling were written on the label and stick on the vials. All samples were sent to School of Public Health, Tehran University of Medical Sciences, where the author identified the specimens using the keys of aquatic insects based on Guide to Aquatic Invertebrate Families of Mongolia 2012 and other relevant systematic keys (18–24). In this study, we used stereo-typed microscope and microscope for identification of samples. The results were recorded on a data sheet based on the order and family and number of its. All of the photos are original.

## Results

Totally, 285 samples were collected belonging to four orders: Diptera, Hemiptera, Ephemeroptera, and Coleoptera (Table 1). Seven families were identified as Tipulidae, Chironomidae, Corixidae, Culicidae, Hydrophilidae and Dytiscidae. The most predominant family was Culicidae (71.2%) belonging to the order Diptera. In the family Culicidae, the species belong to *Culiseta longiareolata* (200 cases) and *Culex hortensis* (3 cases).

**Table 1.** Abundance of some aquatic insects’ larvae collected in the study areas

**Order**	**Family**	**Sample No**	**Percent**
**Ephemeroptera**	Baetidae	25	8.7
**Diptera**	Tipulidae	9	3.2
	Culicidae	203	71.2
	Chironomidae	20	7.1
**Coleoptera**	Dytiscidae	2	1.1
	Hydrophilidae	4	1.6
**Hemiptera**	Corixidae	20	7.1
**Total**		285	100

## Discussion

During study, the specimens were collected via different methods such as D-frame net-collector, standard mosquito dipper (350 ml), Sweep Netting and plastic pipette. A total 285 samples have been grouped in four orders: Diptera, Hemiptera, Ephemeroptera, Coleoptera and the most abundant of them belong to the order Diptera. This order is one of the richest groups of insect and has worldwide distribution with more than 152000 described species based on Biosystematics Database of World Diptera (25). However, in aquatic insect ecosystems, Diptera play an important role and more than half of them belong to this group. The colonies of Diptera are often found in clean and fresh water and in many cases they are the most abundant taxa (26).

In our study, the main family and species of Diptera belong to Culicidae and *Cs. longiareolata* (71.2%). Knight and Stone in 1977 reported that the genus of *Culiseta* consists of 37 species in 7 subgenus which most of them found in the Palearctic and Nearctic Regions (27). In Iran, two subspecies *Allotheobaldia* and *Culiseta* reported up now (28). The subgenus *Allotheobaldia* has only one species (*Cs. longiareolata*) in the world and reported from the Palearctic and Ethiopian Regions, Pakistan and Indian (27). This species reported for the first time by Gusevich in 1943 from north of Iran (30). Then Dow in 1953 reported it from Gorg-Abad, Sharafkhane port and Maraghe in Northwest of Iran (31). *Culiseta longiareolata* larvae usually could be able grow in transient water, without vegetation and mud bottom. Also it collected from rain water pools with shaded and partial sunlight larval habitats. This species has not been reported from artificial habitat until now (32). Azari-Hamidian in 2003 explains this species from Guilan (33). In the same study which carried out by Shayeghi et al. in Isfahan Province, most of the specimens which collected belong to Diptera order (*Cx. theileri*) and their result was very closely to our result (34). Abai et al. (2007) presented the same results in their investigation (35). In study of Shayeghi et al. 2017 in northern of Iran different species of Trichoptera, Ephemeroptera, Plecoptera, Hemiptera and Odonata have been collected and Diptera order was the most prevalent samples (24.5%) (36). In other study was carried out in Markazi provinces, 24 species of aquatic beetles in five families identified (37) and Hydrophilidae is one of the species that we also reported it in our study. Shayeghi et al. in 2015 reported three orders of Plecoptera, Trichoptera and Ephemeroptera from Karaj River (38). The main family in current study belongs to Perlidae (49.7%) (Order: Plecoptera). In Shayeghi study in Jajroud River, five families (Blephariceridae, Simulidae, Hydropsychidae, Baetidae, and Dytiscidae) were identified and the main order belongs to Diptera that is the same as our results (36).

Ostovan et al. (2004) studied on biodiversity and fauna of aquatic insects and beetles in Ardabil and Fars Provinces (39). Also, Atamehr in 2002 and 2004 reported 51 species in 40 genera and 14 families from east Azerbaijan Province (40, 41). Eight orders of aquatic insects includes Ephemeroptera, Odonata, Plecoptera, Hemiptera, Megaloptera, Coleoptera, Trichoptera and Diptera have been reported from Reese Voshell study in USA (42) and we also collected Ephemeroptera, Hemiptera and Diptera in our studies, which is similar to the above results. The second order, which had a high abundance in this study, belonged to Mayfly or Ephemeroptera, (Family: Baetidae). 40 families of mayflies found in worldwide, consisting of about 3330 species (43). The families of mayflies are divided into tree sub-orders, Setisura, Pisciforma and Rechtracheata (44). Mayflies have distributed throughout the world and live in freshwater and sometimes brackish waters on all continents except Antarctica (42). There are different organisms that live in or on mayfly’s body, including a variety of Bacteria, Protozoa, Nematodes (round worms), Cestodes (tape worms) and Trematodes parasite. As well as they act as final hosts for parasites and intermediate hosts, notably for fish parasites (43). In Shayeghi et al. studies in 2017, Mayflies including tree families: Baetidae (91.5%), Heptagenidae (5.5%) and Caenidae (3%) have been collected from rice-field, slow moving river, temporary pond and shallow stream (36). The results of their studies was very similar to our results. Baetidae act as a major component of invertebrate drift in running water (45).

## Conclusion

According to the results, it can be concluded that various species of aquatic insects were collected and identified during study. The main order in study was Diptera. Aquatic insects usually play an important role in the food chain of animal. There are need to do more studies about this important subject. There are some current studies on aquatic insects in different parts of Iran (45–51). Some species of aquatic insects act as predators and play an important role in biological control of vectors as well as bioindicator for aquatic conditions. The authors declare that there is no conflict of interests.
